# The Impact of Aspirin Use on In-Hospital Outcomes and Metastatic Disease in Colorectal Cancer: An Evaluation of the National Inpatient Sample

**DOI:** 10.3390/jcm15103894

**Published:** 2026-05-18

**Authors:** Omar A. Oudit, Temitayo Adebowale, Abdulrahman Atasi, Kibwey Peterkin, Jamal Perry, Chidiebele E. Omaliko, Jamil Shah

**Affiliations:** 1 Department of Internal Medicine, Brookdale University Hospital Medical Center, Brooklyn, NY 11212, USA; abdulrahmanatasi@gmail.com; 2 Department of Biomedical Science, Columbia Irving Medical Center, New York, NY 10032, USA; ta2796@columbia.edu; 3 Department of Palliative Care, Yale University, New Haven, CT 06511, USA; kibwey.peterkin.research@gmail.com; 4 Department of Cardiology, University of Cincinnati, Cincinnati, OH 45219, USA; jcperry908@gmail.com; 5 Department of Gastroenterology, Brookdale University Hospital Medical Center, Brooklyn, NY 11212, USA; comaliko@bhmcny.org; 6 Department of Gastroenterology, The Brooklyn Hospital Center, Brooklyn, NY 11201, USA; jshah2@tbh.org

**Keywords:** aspirin, colorectal cancer, cyclooxygenase, cyclooxygenase, metastatic disease

## Abstract

**Background**: Aspirin, initially recognized for its anti-inflammatory, antipyretic and analgesic properties, holds a prominent role in the treatment of cardiovascular disease. The utility of aspirin in cancer therapeutics has been explored and stratified into COX-dependent and -independent mechanisms. *COX2* gene expression has been demonstrated to be significantly upregulated in colorectal cancer and various other gastrointestinal malignancies including pancreatic, esophageal, and gastric cancer. This study investigates the relationship of aspirin use and outcomes in patients with colorectal cancer. **Methods**: The Nationwide Inpatient Sample (NIS) database from 2017 to 2022 was analyzed for patients age > 18 who were hospitalized for colorectal cancer and its decompensations using ICD-10 diagnostic codes. These patients were further stratified based on the long-term use of aspirin. The principal outcome of this investigation are the odds of in-hospital mortality, with secondary outcomes including odds of pulmonary embolism, portal vein thrombosis, acute kidney injury, septic shock, requiring an ICU level of care and odds of hepatic, pulmonary, gastrointestinal and peritoneal or retroperitoneal metastatic disease. Multivariate logistic regression accounting for hospital and patient characteristics was implemented for analysis, with the Charlson Comorbidity Index used to adjust for coexisting comorbidity burden; a *p*-value (*p*) of <0.05 was considered statistically significant. **Results**: In our analysis of the NIS, 596,160 patients were identified with colorectal cancer and 11.7% (69,750) of this population were identified with long-term use of aspirin. Aspirin use was identified to have a significantly reduced odds of in-patient mortality (adjusted odds ratio) [aOR] 0.530, *p* value < 0.001 95% CI (confidence interval): 0.460–0.617. Patients with aspirin use also demonstrated significantly reduced odds of adverse outcomes and gastrointestinal, hepatic, pulmonary and retroperitoneal/peritoneal metastasis; (aOR 0.606, 95% CI: 0.564–0.653, *p* < 0.001), (aOR 0.628, 95% CI: 0.582–0.678, *p* < 0.001), (aOR 0.676, 95% CI: 0.605–0.755, *p* < 0.001) and (aOR 0.751, 95% CI: 0.685–0.825, *p* < 0.001) respectively. **Conclusions**: In recent years, there has been an alarming increase in incidence of colorectal cancer, particularly amongst younger individuals with increased associated mortality. This mortality increase, albeit alarming, is a driving force for treatment innovation with continual examination of our repertoire of medications for possible repurposed applications. COX2-mediated signaling serves as a key promotor of tumorigenic molecular signaling that directly contributes to tumor cell proliferation, angiogenesis and metastasis in colorectal cancer. Aspirin use and its inhibitory action on COX2 demonstrated a significantly reduced odds of in-hospital mortality. Aspirin use is also associated with significantly reduced odds of developing metastatic disease to the liver, gastrointestinal system, lungs and peritoneum in patients with colorectal cancer. These findings convey that aspirin use reduces the likelihood of in-hospital mortality, major comorbid conditions and of developing metastatic disease as compared to those who do not use aspirin.

## 1. Introduction

The willow bark extract, acetylsalicylic acid, also known as aspirin, was initially recognized for its anti-inflammatory, antipyretic, and analgesic properties in the late 1600s and now holds a prominent role in the treatment of acute coronary syndrome and chronic cardiovascular disease [1]. The leading cause of death across the globe remains cardiovascular disease, closely followed by malignancy. According to the American Cancer Society, in 2022, an estimated 20 million new malignancy cases were diagnosed, with 9.7 million cancer deaths [2,3]. The leading causes of cancer-related mortality in the United States are lung, colorectal, and pancreatic cancer. Despite recent advancements in the treatment of colorectal cancer, it remains a deadly disease with an alarming increase in incidence among younger individuals. This uncontrolled increase in colorectal cancer mortality serves as a driving force for the innovation and development of novel therapeutic treatments. It also stimulates the continual examination of current treatment modalities and our repertoire of medications for possible repurposed applications.

The utility of aspirin in cancer therapeutics has been studied by several groups and stratified into COX-dependent and -independent mechanisms. Cellular *COX2* gene expression is significantly increased in colorectal cancer and various other gastrointestinal malignancies, including esophageal and gastric cancer. Further studies have revealed that COX2 overexpression is also associated with accelerated tumor growth and the induction of molecular processes known to accelerate cancer progression. Aspirin demonstrates several COX-independent molecular interactions with key mitotic regulators of the cell cycle, p53 and various cyclins, and other pathways implicated in tumor carcinogenesis [4]. These interactions interfere with cancer genomics and biochemistry, including cancer cell DNA replication, proliferation, protein expression, and mechanisms of metastasis and angiogenesis [4,5,6]. Prior studies have garnered significant scientific attention with special interest in gaining a mechanistic understanding of how aspirin functions in the treatment and prevention of malignancies, including colorectal cancer. A number of groups have identified aspirin’s association with decreased incidence of and mortality from colorectal cancer [7,8,9,10]. However, these studies are limited by sample size and confounders, including comorbidities, patient demographics, and age. Hence, we conducted this analysis to measure the effects of long-term aspirin use on the likelihood of in-hospital mortality, inpatient outcomes, decompensations, and its association with metastatic disease in those with colorectal cancer on a national scale.

## 2. Materials and Methods

The National Inpatient Sample (NIS), administered by the Healthcare Cost and Utilization Project (HCUP) under the Agency for Healthcare Research and Quality, stands as the largest repository of inpatient hospitalization records in the United States. This database aggregates information from hospitals across 37 states and serves as a dependable resource for estimating disease prevalence and analyzing outcomes. Within the NIS, each hospital stay is anonymized and cataloged as a distinct entry, featuring one primary discharge diagnosis and a maximum of 39 secondary diagnoses, varying according to the data collection year. Each entry contains comprehensive patient demographics (such as age, gender, and race), insurance status, details of primary and secondary procedures (up to 25), hospitalization outcomes, total charges, and length of stay (LOS). 

### 2.1. Inclusion Criteria, Population of Study and Examined Variables

The primary endpoint of our study is comparing the odds of in-hospital mortality between aspirin and non-aspirin using cohorts; while secondary endpoints included the odds of developing septic shock, portal vein thrombosis, pulmonary embolism, acute kidney injury, intensive care unit (ICU) admission and the development of pulmonary, hepatic, gastrointestinal or peritoneal and retroperitoneal metastasis. The 10th version of the international classification of diseases, ICD 10, diagnostic codes were used to identify patients who were greater than 18 years of age who were hospitalized between the years of 2017 to 2022 with a primary diagnosis of colorectal cancer; Supplementary Table S2. Patients were stratified into two groups based on the presence or absence of long-term aspirin use identified during their hospitalization. The final study cohort consisted of 596,160 cases. In this study the primary exposure variable is the use of aspirin in patients with colorectal cancer. Information on variables such as race, gender, age, median income and hospital characteristics including urban versus rural location, bed size and hospital region were also adjusted for during the analysis. The analysis of the comorbidity burden was investigated using the Charleson Comorbidity Index, CCI. The CCI is a well validated clinical index including 19 classes of comorbidities that serves as a clinical prediction tool for a patient’s comorbidity burden.

### 2.2. Statement of Ethics

All data used in this study was captured from the NIS database which represents completely de-identified information. An IRB approval was not required for this study as all patient information is de-identified.

### 2.3. Statistical Analysis

Hospital-level discharge weights provided by NIS were used to generate national estimates. Categorical variables were compared using the chi-square test, whereas an independent sample *t*-test was used for continuous variables. To investigate the effect of defined variables on in-hospital outcomes, univariate logistic regression was performed using a *p*-value threshold of 0.2 for variable selection. Variables meeting this inclusion criterion were subsequently entered into a multivariable logistic regression model for further analysis. Adjusted odds ratios (aORs) were calculated with corresponding 95% confidence intervals (CIs), and statistical significance was defined as a two-tailed *p*-value < 0.05. All analyses were conducted using Stata/MP version 19.5. Modified Poisson regression models with robust standard errors were used as sensitivity models to evaluate the robustness of the primary multivariable logistic regression findings. This approach was selected as an alternative model specification for evaluation of in-hospital mortality and the secondary outcomes evaluated in our study. Analyses were restricted to adult hospitalizations with colorectal cancer stratified by aspirin use. Modified Poisson regression models were constructed for each adverse in-hospital outcome, including pulmonary embolism, portal vein thrombosis, septic shock, intensive care unit stay, acute kidney failure, gastrointestinal, hepatic, pulmonary and peritoneal/retroperitoneal metastasis. The primary exposure variable was aspirin use. All models adjusted for demographic and comorbidity covariates. Robust variance estimation was applied to account for potential misspecification of the Poisson variance structure when modeling outcomes. Model goodness-of-fit was assessed for the multivariable logistic regression model evaluating in-hospital mortality, and all other secondary outcomes in our study, amongst adults hospitalized with colorectal cancer stratified by aspirin use. Model calibration was evaluated using the Hosmer–Lemeshow goodness-of-fit test. Predicted probabilities of in-hospital mortality and all other secondary outcomes were generated from the multivariable logistic regression model, and observations were grouped into 10 quantiles, or deciles, according to their estimated risk. Within each decile of predicted risk, observed events were compared with the number of events expected by the model. The Hosmer–Lemeshow chi-square statistic was then used to assess whether there was evidence of significant disagreement between observed and expected events across risk strata.

## 3. Results

Our study interval from 2017 to 2022 contained 205,215,316 hospitalizations, of which 596,160 adult patients were diagnosed with colorectal cancer, representing the subpopulation in our study. Of these, 69,750 (11.7%) were identified as using aspirin, while 526,410 (88.3%) patients did not use aspirin. Of note, long-term aspirin users were, on average, older than non-aspirin users at 73 and 69 years old, respectively. Further characterization of baseline patient characteristics categorized by the use of aspirin is presented in Table 1.

A greater occurrence of chronic obstructive pulmonary disease, metabolic disorders including obesity and type 2 diabetes mellitus, dyslipidemia, hypertension, and cardiac arrythmias were observed amongst individuals who used aspirin. Interestingly, non-aspirin users were observed to have greater occurrence of ascitic liver disease, cirrhosis, and polysubstance use disorder, including cannabis use and alcohol use disorder; see further characterization in Table 2.

### 3.1. In-Hospital Mortality

Patients with colorectal cancer and aspirin use demonstrated decreased odds of in-hospital mortality, adjusted odds ratio, (aOR) 0.530; *p* value < 0.001 95% CI (confidence interval): 0.460–0.617. Those with colorectal cancer and no aspirin use demonstrated increased odds of in-hospital mortality (aOR) 1.878; *p* value < 0.001 95% CI (confidence interval): 1.62–2.17. The total proportion of mortality amongst patients with colorectal cancer with aspirin use is 1.59% versus 2.78% in non-aspirin users; *p*: <0.00004.

### 3.2. Pulmonary Embolism

Patients with colorectal cancer and aspirin use demonstrated a decreased odds of developing pulmonary embolism, aOR 0.530; *p* value < 0.001 95% CI: 0.431–0.656. Those with colorectal cancer and no aspirin use demonstrated increased odds of developing pulmonary embolism (aOR) 1.88; *p* value < 0.001 95% CI (confidence interval): 1.52–2.32. The total proportion of pulmonary embolism in aspirin users was identified to be 0.77% versus 1.47% in patients with colorectal cancer and no aspirin use; *p*: <0.0001.

### 3.3. Portal Vein Thrombosis

The aOR of developing portal vein thrombosis for patients with colorectal cancer and aspirin use is 0.500; *p* value < 0.007 95% CI: 0.306–0.831. Those with colorectal cancer and no aspirin use demonstrated increased odds of developing portal vein thrombosis (aOR) 1.98; *p* value < 0.006 95% CI (confidence interval): 1.20–3.26. The total proportion of portal vein thrombosis in aspirin users was identified to be 0.12% as compared to 0.35% in non-aspirin users; *p*: <0.00004.

### 3.4. Septic Shock

Patients with colorectal cancer and aspirin use demonstrated decreased odds of developing septic shock, aOR 0.380; *p* value < 0.001 95% CI: 0.301–0.487. Those with colorectal cancer and no aspirin use demonstrated increased odds of septic shock (aOR) 2.61; *p* value < 0.001 95% CI (confidence interval): 2.06–3.32. The total proportion of septic shock in aspirin users was identified to be 0.55% versus 1.39% in patients with no aspirin use; *p*: <0.00007.

### 3.5. ICU Stay

Patients with colorectal cancer and aspirin use demonstrated decreased odds of requiring an ICU admission, aOR 0.640; *p* value < 0.001 95% CI: 0.570–0.717. Those with colorectal cancer and no aspirin use demonstrated increased odds of requiring an ICU level of care (aOR) 1.56; *p* value < 0.001 95% CI (confidence interval): 1.39–1.75. The total proportion of critical care escalation as determined by in-hospital ICU admission was measured to be 3.71% in non-aspirin users vs 2.67% in patients with aspirin use; *p*: <0.00002.

### 3.6. Acute Kidney Injury, AKI

**Figure 1:**
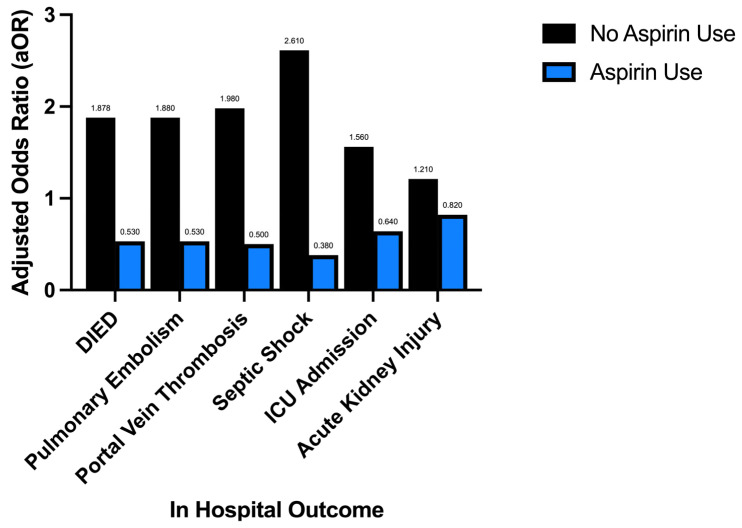
Effect of aspirin vs. no aspirin use on in-hospital outcomes in colorectal cancer.

The aOR of developing AKI for patients with colorectal cancer and aspirin use is 0.82; *p* value < 0.001 95% CI: 0.779–0.871. Those with colorectal cancer and no aspirin use demonstrated increased odds of developing an AKI (aOR) 1.21; *p* value < 0.001 95% CI (confidence interval): 1.15–1.28. The total proportion of acute kidney failure in aspirin users was identified to be 13.72% versus 12.49% in patients with no aspirin use; *p*: <0.0001. The unadjusted odds ratio of developing an AKI is 1.11 CI: 1.05–1.17 *p*: <0.001, Supplementary Table S1, and although the identified proportion of AKI is slightly greater than that in those without aspirin use, the adjustment for demographic factors and confounders reveals a statistically significant decreased odds of developing an AKI in those with colorectal cancer and aspirin use as compared to those without aspirin use. This reflects the absence of adjustment for demographic factors and confounders that initially seem to demonstrate increased odds of AKI with aspirin use however once accounted for convey statistically preserved, *p* < 0.05, decreased odds of AKI in those with aspirin use and colorectal cancer as compared to those with colorectal cancer without aspirin use; further demonstrated in Table 3 and Figure 1.

Aspirin use significantly reduces the odds of in-hospital mortality and the occurrence of major clinical comorbidities including pulmonary embolism, portal vein thrombosis, acute kidney injury, the onset of septic shock, and ICU-level care. Those with colorectal cancer and no aspirin use demonstrated significantly increased odds of in-hospital mortality and the occurrence of the same major clinical comorbidities.

Aspirin use in those with colorectal cancer is associated with a decreased likelihood of in-hospital mortality and the development of major secondary complications, including pulmonary embolism, portal vein thrombosis, acute kidney injury, the onset of septic shock, and ICU-level care. Those with colorectal cancer and no aspirin use demonstrate significantly increased odds of in-hospital mortality and of developing the same major comorbid conditions associated with colorectal malignancies.

### 3.7. Gastrointestinal Metastasis

Patients with colorectal cancer and aspirin use demonstrated a decreased odds of developing gastrointestinal metastasis, aOR 0.606; *p* value < 0.001 95% CI: 0.564–0.653. Those with colorectal cancer and no aspirin use demonstrated increased odds of developing gastrointestinal metastasis (aOR) 1.64; *p* value < 0.001 95% CI (confidence interval): 1.53–1.77. The total proportion of gastrointestinal metastasis in aspirin users was identified to be 15.47% versus 23.86% in non-aspirin users in patients with colorectal cancer; *p*: <0.0001.

### 3.8. Hepatic Metastasis

Patients with colorectal cancer and aspirin use demonstrated a decreased odds of developing hepatic metastasis, aOR 0.628; *p* value < 0.001 95% CI: 0.582–0.678. Those with colorectal cancer and no aspirin use demonstrated increased odds of developing hepatic metastasis (aOR) 1.59; *p* value < 0.001 95% CI (confidence interval): 1.47–1.71. The total proportion of hepatic metastasis in aspirin users was identified to be 10.22% versus 16.71% in non-aspirin users in patients with colorectal cancer; *p*: <0.0001.

### 3.9. Pulmonary Metastasis

Patients with colorectal cancer and aspirin use demonstrated a decreased odds of developing pulmonary metastasis, aOR 0.676; *p* value < 0.001 95% CI: 0.605–0.755. Those with colorectal cancer and no aspirin use demonstrated increased odds of developing pulmonary metastasis (aOR) 1.47; *p* value < 0.001 95% CI (confidence interval): 1.32–1.65. The total proportion of pulmonary metastasis in aspirin users was identified to be 3.26% versus 5.55% in non-aspirin users in patients with colorectal cancer; *p*: <0.0001.

### 3.10. Peritoneal and Retroperitoneal Metastasis

**Figure 2:**
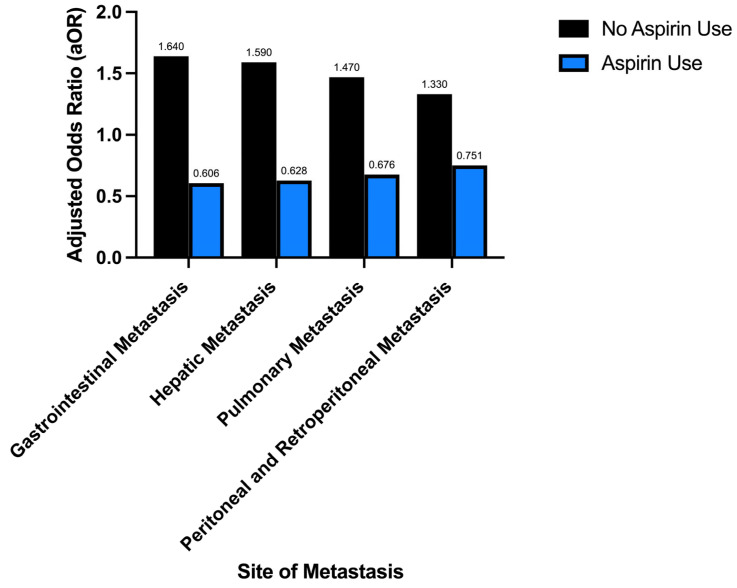
Aspirin use decreases the likelihood of metastatic disease in colorectal cancer.

Patients with colorectal cancer and aspirin use demonstrated a decreased odds of developing peritoneal and retroperitoneal metastasis, aOR 0.751; *p* value < 0.001 95% CI: 0.685–0.825. Those with colorectal cancer and no aspirin use demonstrated increased odds of developing peritoneal and retroperitoneal metastasis aOR 1.33; *p* value < 0.001 95% CI (confidence interval): 1.21–1.46. The total proportion of peritoneal and retroperitoneal metastasis in aspirin users was identified to be 5.21% versus 8.03% in non-aspirin users in patients with colorectal cancer; *p*: <0.0001; further demonstrated in Table 4 and Figure 2.

Aspirin use in those with colorectal cancer is associated with significantly decreased odds of gastrointestinal, hepatic, pulmonary, peritoneal, and retroperitoneal metastasis. Those with colorectal cancer and no aspirin use demonstrate significantly increased odds of developing metastatic disease at these sites.

Aspirin use in those with colorectal cancer demonstrates significantly decreased odds of gastrointestinal, hepatic, pulmonary, peritoneal, and retroperitoneal metastasis. Those with colorectal cancer and no aspirin use demonstrate significantly increased odds of developing metastatic disease at these sites. Aspirin use in those with colorectal cancer therefore potentially affects the likelihood of metastatic disease.

## 4. Discussion

In our evaluation of the effects of aspirin in 596,160 cases of colorectal cancer, we have identified a statistically significant reduction in in-hospital mortality amongst long-term users of aspirin compared to non-aspirin users. These findings are supported by those in several other groups, such as in a pooled analysis of two cohort studies that compared rates of development of colorectal cancer in those who used aspirin versus no aspirin use. Aspirin use was associated with a significantly reduced likelihood of developing colorectal cancer compared to no aspirin use [11]. The researchers subsequently identified a time-dependent relationship between the incidence of colorectal cancer and total duration of exposure to aspirin. Specifically, patients with longer consecutive use of aspirin demonstrated lower incidences of colorectal cancer than non-aspirin users; the study involved 14,023 patients with average aspirin exposure time of six years and a median follow-up time between the aspirin and control groups of 18.3 years [11]. Another study reported a similar reduction in the rate of colorectal-cancer-related mortality amongst patients with concurrent aspirin use [12], and a prospective cohort study of 1279 men and women diagnosed with stage one, two, or three colorectal cancer with a median follow-up time of 11.8 years found that regular aspirin use is significantly associated with decreased colorectal-cancer-specific and overall mortality [7].

While these findings suggest a survival benefit of aspirin use in colorectal cancer, the precise biological mechanisms by which aspirin may exert these protective effects against colorectal cancer and the molecular events that drive its progression remain largely unknown. The targets of aspirin, cyclooxygenase one and two (COX1 and COX2), and their expression profiles in colorectal malignancies have been evaluated by several groups [13,14,15]. Cancer cells derived from these tissues have demonstrated differential protein expression of both COX1 and COX2, with significant elevations of COX2 enzyme levels [13,14,15]. Increased COX2 expression has been found to promote tumor cell growth and to affect the structural integrity of mucosal and vascular tissue through molecular changes that drive and promote chronic tissue inflammation. COX2 has also been identified as an integral promoter of the pathophysiology in colorectal malignancies [13,14,15]. It is well documented in the scientific and clinical literature that states of chronic inflammation are known drivers of malignancy through the increased production of inflammation-induced reactive oxygen species (ROS). Persistent proinflammatory signaling induces prolonged states of oxidative stress and DNA mutations, both of which contribute to tissue carcinogenesis, particularly in colorectal tissue [16,17]. Acetate, a principal breakdown product of aspirin, has been implicated in maintaining the integrity of colonic epithelium [18]. Alongside other short chain fatty acids, aspirin-derived acetate holds promise in enhancing the sensitivity of colorectal cancer cells to chemo- and radiation therapy [18]. The anti-inflammatory properties of aspirin, through the decreased generation of COX1 and COX2 enzyme products, may be a key contributor to the observed decrease in in-hospital mortality identified in our study.

Despite the well-known anti-inflammatory effects driven by cyclooxygenase inhibition, aspirin has been demonstrated to exert protective antineoplastic properties. Several studies of the potential role of aspirin in the treatment of colorectal cancer have stratified the mechanisms into COX-dependent and -independent mechanisms. Loss of function (LOF) mutations of the tumor suppressor *TP53* gene, encoding the p53 protein, are a known inciting molecular event leading to several malignancies. The LOF of *TP53* has been characterized in approximately 50–75% of patients with colorectal malignancies [19]. p53 primarily serves as a cell cycle checkpoint inhibitor in the G1-S phase and G2-M phase [19]. When activated, p53 mediates apoptosis in atypical cells, ultimately protecting the organism from the proliferation of cells with damaged DNA and dysregulated cellular growth signaling [19]. Aspirin has been shown to directly acetylate and activate p53 proteins in vivo and vitro in several gastrointestinal malignancies [20]. These molecular and genetic derangements therefore remain molecular targets of interest in the augmentation of colorectal cancer therapeutics. Recent studies reveal a growing number of patients with colorectal cancer phenotypes with greater amounts of resistance to current chemotherapy treatment [21,22,23]. This highlights the need for innovation and a re-evaluation of current medication regimens in the managing of this disease. This COX-independent mechanism may explain the decreased incidence of colorectal cancer and mortality in patients who use aspirin observed by several other groups. Alongside the anti-malignancy properties of aspirin are the clinically significant benefits of aspirin use in mitigating the development of clinical complications noted to significantly enhance morbidity and mortality.

Our study also demonstrates a decreased rate of developing thrombotic events such as pulmonary emboli and deep vein thromboses (DVTs). Malignancies are well-known drivers of hypercoagulable states due to local and systemic deregulatory processes affecting vascular structural integrity and causing pathological derangements of the clotting cascade. Colorectal malignancies have been found to have significantly elevated rates of thrombotic events when compared to other malignancies [24,25,26]. Our data demonstrates statistically significant decreased odds of developing several thrombotic conditions, all of which carry significantly increased risk of mortality and worsened outcomes. Although aspirin does not demonstrate great capacity to dissolve and treat active thrombosis, it is significantly associated with the ability to decrease the incidence of these events. The suppression of COX1 activity decreases the platelet production of thromboxane A2 (TxA2), a key mediator of platelet activation and aggregation [27,28]. TxA2 is enzymatically synthesized from COX1 activity on arachidonic acid within platelets. Once synthesized and released by activated platelets, TxA2 acts as a potent secondary messenger augmenting further downstream platelet activation, aggregation, and vasoconstriction. Aspirin inhibits COX1 activity and, therefore, TxA2 synthesis, preventing the activation of the TxA2 receptor on other platelets and dampening the downstream procoagulant signaling cascade [27,28]. Aspirin also mediates the downregulation of tissue factors, the formation of thrombin, and, by extension, thrombin-coordinated downstream coagulation reactions—all important components of a mature and stable thrombus [29]. This suggests a shielding effect of aspirin use against the development of thrombotic disorders in a particularly high-risk population of patients. This poses an important consideration for the potential prophylactic application of aspirin against the development of such thromboses in patients with colorectal malignancies. Although venous thrombotic events of distal lower extremity vasculature and pulmonary emboli occur more commonly, less frequent events such as portal vein thrombosis are associated with disproportionately increased morbidity and mortality. This includes the development of venous thromboembolisms (VTEs), DVTs, and pulmonary emboli; however, this also extends to more rare sites of thrombosis such as the portal vein. In patients with colorectal cancer, the formation of portal vein thrombosis does represent a rare event, with an estimated incidence rate of less than 1% [30,31]. Although rare, portal vein thrombosis is associated with a statistically significant doubled odds of mortality in patients with colorectal cancer [32]. Our findings of decreased odds of developing portal vein thrombosis (aOR: 0.500; *p*-value: <0.007; 95% CI: 0.306–0.831) with aspirin use highlight an application for this medication in the prevention of a rare but deadly and costly complications of colorectal cancer.

These observations provide strong support for the antithrombotic and vascular benefit of aspirin use in colorectal cancer; however, the clinical application of aspirin and its relationship with rates of metastases remain poorly understood. COX2 has been identified as a key promoter of malignant cell growth in various kinds of cancers [33,34,35]. Several groups have identified increased COX2 expression levels in several gastrointestinal malignancies, including colorectal cancer [33,34,35]. The overexpression of COX2 in colorectal malignancies was identified as a significant predictor of poor prognosis and increased mortality. Various studies have investigated the relationship between the overexpression of COX2 and tumorigenesis, revealing that COX2 is an important driver of advanced-stage tumor development via the regulation of underlying mechanisms involving angiogenesis, proliferation, the inhibition of apoptosis, and metastasis [33,34,35,36,37,38]. A known requisite for tumor progression involves angiogenesis and its regulation of the vascular delivery of nutrients, tumor cell growth, secondary tissue invasion, and metastasis. Indeed, COX2 activity has been identified as a significant promoter of angiogenesis in colorectal cancer through the upregulation of critical pro-angiogenic factors, including vascular endothelial growth factor (VEGF), platelet-derived growth factor beta (PDGFRß), fibroblast growth factor (bFGF) and bFGF receptor, endothelin 1, nitric oxide synthase, and transforming growth factor beta (TGFB) [33,34,35,36,37,38]. Collectively, these studies identify COX2 overexpression as a substantial regulator of the cellular and molecular processes underlying tumor progression, and it remains a molecular target of interest in colorectal cancer therapeutics. This is of particular interest given recently identified chemotherapy resistance amongst patients with colorectal cancer undergoing treatment where the recurrence of tumors and chemotherapy resistance remain leading causes of poor prognosis [39,40,41].

**Figure 3:**
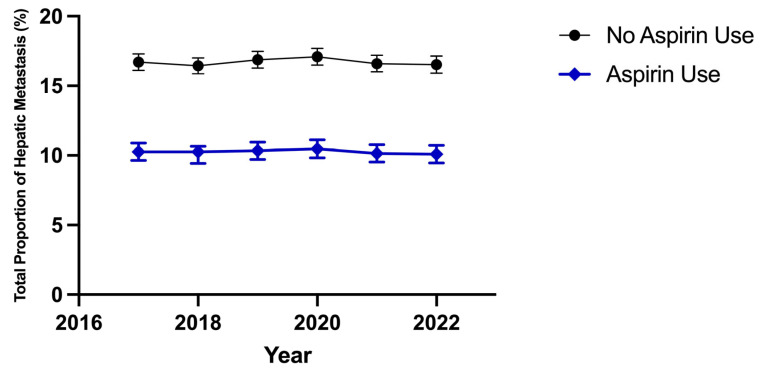
Aspirin use is associated with reduced proportion of hepatic metastasis in colorectal cancer.

The effects of anti-platelet agents, primarily aspirin, on the incidence of metastasis have been evaluated in a limited capacity by some other groups. Several meta-analyses of large-scale randomized control studies have identified a significant decrease in the incidence of metastasis to secondary sites in patients with colorectal cancer who used aspirin compared to controls [42]. The improved survival benefit of aspirin use in those with colorectal cancer has been found to share a relationship with the expression levels of human leukocyte antigen (HLA) type 1, suggesting an immunological component of the anti-metastatic activity of aspirin [43]. This study demonstrates that platelet-derived TXA2 suppresses T-cell immunity to cancer metastasis through the activation and upregulation of a T-lymphocyte-specific pathway that requires a guanine exchange factor protein named ARHGEF1. This was further evaluated by restricting the total pool of TXA2 through aspirin use and the downregulation of COX1 expression, which was shown to reduce rates of metastasis through a novel mechanism involving reduced T-cell expression levels of ARHGEF1 and signaling levels of TXA2 [43]. Correspondingly, it has also been found that ARHGEF1 knockout mice injected with melanoma cell lines demonstrated significantly reduced rates of metastasis, providing further support for the role of aspirin use in enhancing T-cell-dependent, anti-tumor, and anti-metastatic effects [43]. These findings provide mechanistic support for the observed decreased odds of metastatic disease in patients with colorectal cancer and aspirin use, as identified in our study. Given the venous drainage of the colon, the liver remains the most common site of metastasis in colorectal cancer. This study demonstrates that aspirin use is associated with a consistent decrease in observed proportion of hepatic metastasis in those with colorectal cancer and aspirin use compared to those with colorectal cancer and no aspirin use (depicted in Figure 3). The associated occurrence of hepatic metastasis in colorectal cancer with aspirin use in 2017 was 10.26% (CI: 9.63–10.89%, *p*: <0.0001) and in 2022 was 10.09% (CI: 9.46–10.72%, *p*: <0.0001), and the associated occurrence of hepatic metastasis in colorectal cancer patients with no aspirin use in 2017 was 16.7% (CI: 16.1–17.29%, *p*: <0.0001) and in 2022 was 16.52% (CI: 15.9–17.14%, *p*: <0.0002). These findings are supported by those conveyed in another observational retrospective study where aspirin use in colorectal cancer was identified to be associated with a decreased odds of GI and non-GI metastasis [44]. Our findings uniquely diverge from the previously noted by designing a more specific anatomical characterization based on the biologically informed gradient of greatest probability metastatic sites such as the liver and lungs to less likely sites including other gastrointestinal organs and the peritoneum/retroperitoneum. This stratification provides a more refined evaluation of potential associated effects of aspirin use on site of metastasis in those with colorectal cancer. A randomized controlled study and editorial article have evaluated and commented on the effect of aspirin use on colorectal cancer recurrence in those with aspirin use and the likely usefulness of aspirin as an adjunctive treatment in colorectal cancer [45,46]. The randomized double blinded placebo study, the ALASCCA trial, demonstrated a reduced incidence of colorectal cancer recurrence in those with aspirin use compared to placebo, particularly in those with a specific molecular genotype identified as specific exon 9 and 20 mutations in PIK3CA [45]. Notably, they also identified a similar signal amongst those with other PI3K pathway-specific mutations in colorectal cancer [45]. The editorial piece synthesized varying levels of evidence with the overall deduction that there is mechanistic plausibility between aspirin’s effects and improved outcomes in those with colorectal cancer however they are likely most pronounced in certain molecular phenotypes as opposed to a uniform benefit in all individuals with colorectal cancer [46]. These studies provide support for the findings conveyed in our study, however, highlight the continued need of further prospective investigation of the colorectal-cancer-specific, survival and in-hospital outcomes with aspirin use. Further prospective studies are necessary to clarify the directionality of the observed associations and to determine whether these findings have true temporal relationships to aspirin use and if there is any cross-sectional element. Longitudinal and prospective study designs are required to validate these findings, assess temporal sequencing, and more precisely characterize the mechanisms underlying the observed associations.

Aspirin use in those with colorectal cancer demonstrated significantly decreased odds of developing hepatic metastatic disease consistently throughout the study interval of 2017–2022. Those with colorectal cancer and no aspirin use consistently demonstrated significantly increased odds of developing metastatic disease of the liver. Aspirin use in those with colorectal cancer suggests a decreased likelihood of hepatic metastatic events identified as compared to those with colorectal cancer and no aspirin use. Total proportion of hepatic metastasis in colorectal cancer with aspirin use 2017: 10.26% (CI: 9.63–10.89%, *p*: <0.0001) and in 2022: 10.09% (CI: 9.46–10.72%, *p*: <0.0001); total proportion of hepatic metastasis in colorectal cancer with no aspirin use in 2017: 16.7% (CI: 16.1–17.29%, *p*: <0.0001) and in 2022: 16.52% (CI: 15.9–17.14%, *p*: <0.0002).

In addition to colorectal-cancer-induced vascular and gastrointestinal pathophysiology, this malignancy is associated with derangements in immune cell function that precipitate infectious complications driving clinical decompensation. Colorectal cancer, like other malignancies, leads to several immune dysregulations that both suppress and alter the immune system in ways that promote its growth. One such mechanism involves colorectal cancer cells inducing the dysregulation of immunosuppressive molecules such as interleukin 10 (IL10), TGFB, MUC5A, and MUC1 [47,48,49,50,51,52,53,54,55]. These signaling molecules normally play a key role in the regulation of immune cell responses in normal physiology; however, their secretion in a dysregulated, constitutive fashion allows for dampening of the host immune response at both local and systemic scales, supporting rapid tumor growth [47,48,49,50,51,52,53,54,55]. Another major consequence of this immune dysregulation is the host’s inability to properly fight and clear infections. Infections impose a significant clinical burden on patients with malignancies and often precipitate major complications and a worse prognosis [56]. Several recent studies have found that up to 60% of malignancy-related mortality is derived both directly and indirectly from the incidence of infections [56,57]. The nature of cancer treatment, including chemo- and radiation therapy in colorectal cancer, greatly predisposes patients to the development of infections. If infectious disease occurs, the patient’s treatment necessitates complete cessation of cancer treatment, which directly impinges on the treatment course.

This immune dysfunction frequently precipitates infections, leading to systemic hemodynamic compromise that drives multiorgan involvement, necessitating an ICU level of care. AKI represents a major complication in those with malignancies, with recent evaluations indicating an increased frequency of AKI, particularly within the first year of diagnosis, with a significant proportion of those affected becoming critically ill [58]. Acute renal failure in malignancy patients is often attributed to rapid cellular turnover from aggressive cancer or the development of tumor lysis syndrome in the setting of tumor cell destruction from chemotherapy or radiation therapy. Our findings demonstrate that aspirin use was indeed associated with significantly decreased odds of developing septic shock—hemodynamic decompensation in the setting of any infection—in patients with colorectal cancer. We also demonstrated that aspirin use was associated with a concurrent significantly decreased odds of requiring ICU-level care and of developing an AKI. Other groups have identified a significant incidence of AKI in patients with colorectal cancer who undergo varying treatment modalities [58,59,60]. This is another potential application of aspirin use in patients with colorectal cancer. These complications are known to significantly increase both mortality and morbidity in patients with colorectal cancer. Our data supports the notion that aspirin use may help prevent further decompensation in patients admitted for clinical manifestations related to colorectal cancer or its progression. These in-hospital complications influence short-term mortality, but their effects extend well beyond this and have longer-lasting implications for the utilization of healthcare resources and patients’ quality of life.

Aspirin’s therapeutic potential resides not only in its clinical benefits but also with its decreased likelihood of major ICU admissions. The ICU, although a lifesaving escalation of care, is not without complications. Several single- and multi-center analyses evaluated the factors that influence the development of post-intensive care syndrome (PICS) [61,62,63,64]. The incidence of septic shock, hypotension, the use of vasoactive medications, age at time of admission, and infections were found to significantly increase the risk of PICS and subsequently decrease quality of life [61,62,63,64]. Our findings support the fact that aspirin use in colorectal cancer not only provides in-hospital clinical benefits but is also shown to protect patients from severe clinical decompensations with significant likelihood of negatively impacting their quality of life. Aspirin use therefore also serves to minimize healthcare resource utilization, further decreasing total hospital length of stay and overall hospital charges, thus translating to decreased financial burden on the healthcare system.

Our observations provide compelling support for the multifaceted benefits of aspirin use in patients with colorectal cancer; however, several limitations must also be considered when evaluating this data. We conducted a retrospective analysis of the NIS, where the study population was selected for inclusion by the user and is therefore subject to selection bias. To mitigate this effect, standardized selection protocols using the ICD 10 coding system were implemented to identify patients with long-term aspirin use and colorectal cancer, the accuracy of which is reliant on the correct and consistent entry of appropriate ICD 10 codes during patient encounters. This is subject to various degrees of fluctuation in different hospital systems with varying policies. Although vast in nature, the NIS database does not capture information on indications for medications, the total duration of therapy, or dosage. Also, the NIS does not directly stratify malignancy diagnoses based on cancer staging and is therefore not able to directly capture unique cancer staging or to account for unmeasured confounders. Unique ICD 10 codes were used to identify patients with colorectal cancer, long-term use of aspirin, and in-hospital outcomes in our study. This was performed to maximize the accuracy of data collection, minimize user error when designing codes, and ensure the reproducibility of our study. Our study has successfully investigated the effects of aspirin exposure in patients with colorectal cancer while also integrating the impact of patient demographic factors and age into the analysis. The findings in our study are supported by high statistical power. Our study utilizes data obtained and maintained by the HCUP over an interval of six years, therefore conferring a large enough sample size that counterbalances several of these limitations and supports the larger power of our study findings in comparison to several other single-center or smaller multicenter studies. Our study shows a statistically significant decreased likelihood of in-hospital mortality and adverse secondary outcomes, suggesting a potentially beneficial effect of aspirin. However, the net clinical gain from its use in those with colorectal cancer remains contingent on diligent patient selection with individualized risk stratification. Integrating our study findings with these considerations emphasizes the importance of a well-balanced patient-centered approach in which the potentially beneficial advantages of aspirin are carefully tempered by the inherent bleeding risk associated with its use and is conducted with guideline-directed clinical decision making.

## 5. Conclusions

Fundamentally, our study demonstrates that patients with colorectal cancer who use aspirin demonstrate a significant reduction in in-hospital mortality and known decompensations and comorbidities that drive increased mortality and healthcare resource utilization. Aspirin use is also associated with a significantly decreased odds of developing metastatic disease in those with colorectal cancer compared to those who do not use aspirin. Given the retrospective nature of NIS analysis, future prospective longitudinal studies must be performed to further investigate the relationship between aspirin use and colorectal cancer incidence, outcomes and impact on metastatic disease. Considering the ubiquitous availability and relatively minimal cost of aspirin, these findings both reveal and support the multifaceted benefits of its use in these patients, and future prospective research needs to be performed to analyze the role of aspirin in both the primary prevention of malignancies and as an adjunctive treatment agent while weighing its benefits against inherent bleeding risk.

## Figures and Tables

**Table 1:** Characterization of patients identified by aspirin use.

Characteristic	No Aspirin Use	Aspirin Use	*p*-Value
Age 18–44	5.8	0.6	<0.001
Age 45–64	37.1	21.1	<0.001
Age > 64	53.9	75.7	<0.001
Male	50.2	57.2	<0.001
Female	49.8	42.8	
White	69.9	77.6	<0.001
Black	13.0	11.1	
Hispanic	9.7	6.1	
Asian/Pacific Islander	3.9	2.8	
Native American	0.5	0.4	
Other race	3.0	2.2	
Income quartile 1 (lowest)	27.1	27.5	<0.001
Income quartile 2	26.0	27.4	
Income quartile 3	24.4	24.3	
Income quartile 4 (highest)	22.6	20.8	
Non-teaching hospital	26.1	27.0	0.064
Teaching hospital	73.9	73.0	
Rural hospital	8.4	8.9	0.052
Urban hospital	91.6	91.1	
Northeast	19.0	15.9	<0.001
Midwest	21.2	27.0	
South	39.9	39.4	
West	19.9	17.7	
Small bed size	19.5	19.8	0.041
Medium bed size	28.4	29.5	
Large bed size	52.0	50.8	

**Table 2:** Patient comorbidities characterized by aspirin use.

Comorbidity	No Aspirin Use	Aspirin Use	*p*-Value
COPD	10	15.1	<0.001
Asthma	4.8	4.5	0.129
Obesity	16.2	20.1	<0.001
Type 2 diabetes	22.5	37.6	<0.001
Hypertension	56.2	81.6	<0.001
Hypothyroidism	10.4	13.7	<0.001
Hyperthyroidism	0.42	0.38	0.518
Alcohol use disorder	2.4	2	0.003
HIV	0.28	0.14	0.002
Ventricular tachycardia	1	1.4	<0.001
Ventricular fibrillation	0.093	0.057	0.175
Atrial fibrillation	12.1	17	<0.001
Malnutrition	14.3	10.3	<0.001
Cannabis use disorder	0.86	0.56	<0.001
Dyslipidemia	33.7	63.1	<0.001
Distal DVT	0.35	0.31	0.460
Rheumatoid arthritis	1.3	1.5	0.003
Abnormal electrolytes	26.4	25.6	0.034
Systolic heart failure	0.94	1.19	0.003
Mental disorder	17.6	17.9	0.305
Seizure	1.8	1.8	0.798
Valvular disease	2.7	5.1	<0.001
Cirrhosis	1.8	1.7	0.286
Ascites	6.2	3.6	<0.001

**Table 3:** Multivariate logistic regression analysis examining the relationship of aspirin and no aspirin use on outcomes in colorectal cancer.

Outcomes	No Aspirin Use	95% Confidence Interval	*p*-Value	Aspirin Use	95% Confidence Interval	*p*-Value
DIED	1.878	1.62–2.17	<0.001	0.53	0.460–0.617	<0.001
Pulmonary embolism	1.88	1.52–2.32	<0.001	0.53	0.431–0.656	<0.001
Portal vein thrombosis	1.98	1.20–3.26	<0.007	0.5	0.306–0.831	<0.006
Septic shock	2.61	2.06–3.32	<0.001	0.38	0.301–0.487	<0.001
ICU admission	1.56	1.39–1.75	<0.001	0.64	0.570–0.717	<0.001
Acute kidney injury	1.21	1.15–1.28	<0.001	0.82	0.779–0.871	<0.001

Aspirin use decreases the likelihood of in-hospital mortality and the development of clinical comorbidities.

**Table 4:** Multivariate logistic regression analysis examining the relationship of aspirin and no aspirin use on the likelihood of metastasis in colorectal cancer.

Outcomes	No Aspirin Use	95% Confidence Interval	*p*-Value	Aspirin Use	95% Confidence Interval	*p*-Value
Gastrointestinal metastasis	1.64	1.53–1.77	<0.001	0.606	0.564–0.653	<0.001
Hepatic metastasis	1.59	1.47–1.74	<0.001	0.628	0.582–0.678	<0.001
Pulmonary metastasis	1.47	1.32–1.65	<0.001	0.676	0.605–0.755	<0.001
Peritoneal and retroperitoneal metastasis	1.33	1.21–1.46	<0.001	0.751	0.685–0.825	<0.001

Aspirin use decreases the likelihood of metastasis in colorectal cancer.

## Data Availability

The National Inpatient Sample, NIS, represents the largest de-identified database containing information on various in-hospital outcomes and is maintained by the healthcare cost and utilization project, HCUP; all information contained within NIS database files are uniquely represented and validated by HCUP to ensure accuracy of the information’s content and its origin. Due to privacy and federal regulations governing the maintenance and protected distribution of HCUP data used for analysis in this study direct distribution of this data is federally prohibited. All HCUP data used for analysis in this study is available for access through the following link following engagement with and completion of HCUP mandated registration and HCUP data handling training processes. https://hcup-us.ahrq.gov/team/NationwideDUA.jsp, accessed on 18 April 2025.

## Supplementary Materials

The following supporting information can be downloaded at: https://www.mdpi.com/article/10.3390/jcm15103894/s1, Table S1: Unadjusted odds ratios of all study outcomes; Table S2: Populations and Outcomes ICD10 codes.
