# Supplementary material for: The Impact of Aspirin Use on In-Hospital Outcomes and Metastatic Disease in Colorectal Cancer: An Evaluation of the National Inpatient Sample
Source: J Clin Med. 2026 May 18;15(10):3894. doi: 10.3390/jcm15103894 (PMC13206795; doi:10.3390/jcm15103894)
Supplement: Supplementary file 1 [file jcm-15-03894-s001.zip › jcm-4283977-supplementary.pdf]

Supplementary Table S1: Unadjusted odds ratios of all study outcomes

Outcome	Aspirin Use (OR [95% CI])	P Value	No Aspirin Use (OR [95% CI])	P Value
**In-hospital mortality (DIED)**	0.56 (0.49–0.65)	<0.001	1.78 (1.54–2.05)	<0.001
**Acute kidney failure**	1.11 (1.06–1.17)	<0.001	0.90 (0.85–0.95)	<0.001
**Pulmonary embolism**	0.52 (0.43–0.64)	<0.001	1.91 (1.56–2.33)	<0.001
**Portal vein thrombosis**	0.34 (0.21–0.55)	<0.001	2.94 (1.81–4.79)	<0.001
**Septic shock**	0.40 (0.32–0.50)	<0.001	2.52 (2.00–3.17)	<0.001
**ICU admission**	0.71 (0.64–0.80)	<0.001	1.40 (1.26–1.57)	<0.001
**Hepatic metastases**	0.57 (0.54–0.60)	<0.001	1.76 (1.66–1.87)	<0.001
**Gastrointestinal metastases**	0.58 (0.56–0.61)	<0.001	1.71 (1.63–1.80)	<0.001
**Pulmonary metastases**	0.57 (0.52–0.63)	<0.001	1.74 (1.58–1.92)	<0.001
**Peritoneal/retroperitoneal metastases**	0.63 (0.58–0.68)	<0.001	1.59 (1.47–1.72)	<0.001

Supplementary Table S2: Populations and Outcomes ICD10 Codes

Outcome/population	ICD10 Code(s)
Long-term aspirin use	Z79.82
colorectal cancer	C18, C19
Pulmonary Embolism	I26
Portal Vein thrombosis	I81
Septic shock	R65.21
ICU level of care	3E030XZ, 3E033XZ, 3E040XZ, 3E043XZ, 3E050XZ, 3E053XZ, 3E060XZ, 3E063XZ, 5A1935Z, 5A1945Z, 5A1955Z, 5A0935Z, 5A0945Z, 5A0955Z, 5A09B5K, 5A09D5K, 5A09C5K, 06HM33Z, 06HM43Z, 06HN33Z, 06HN43Z, 05HM33Z, 05HM43Z, 05HN33Z, 05HN43Z, 05H533Z, 05H543Z, 05HB33Z, 05HB43Z, 05HB43Z, 05HC43Z, 05H933Z, 05H943Z, 05HA33Z, 05HA43Z
Acute kidney injury	N17
Gastrointestinal Metastasis	C78
Hepatic Metastasis	C78.7
Pulmonary Metastasis	C78.0
Peritoneal and retroperitoneal Metastasis	C78.6
